# Supplementary material for: Anaerobic oxidation of methane supports a minimal microbial community in a subsurface biofilm at Ginsburg mud volcano
Source: ISME Commun. 2026 Mar 23;6(1):ycag072. doi: 10.1093/ismeco/ycag072 (PMC13109099; doi:10.1093/ismeco/ycag072)

**Figure S1**. Maximum-likelihood phylogenomic tree based on 105 MAGs from the class *Syntrophoarchaeia*, showing the presence/absence mcr genes, archaeal lipid biosynthesis genes and synteny analysis of genomic regions harboring mcr operons across representative MAGs. ANME of interest clustering closely with ANME-1b and JAFNKG01 sp. 030602585. GGPP: geranylgeranyl diphosphate synthase; GGGP: geranylgeranylglyceryl phosphate synthase; DGGGP: digeranylgeranylglyceryl phosphate synthase; car-S: carboxy-SAM synthase; Tes: tetraether synthase.

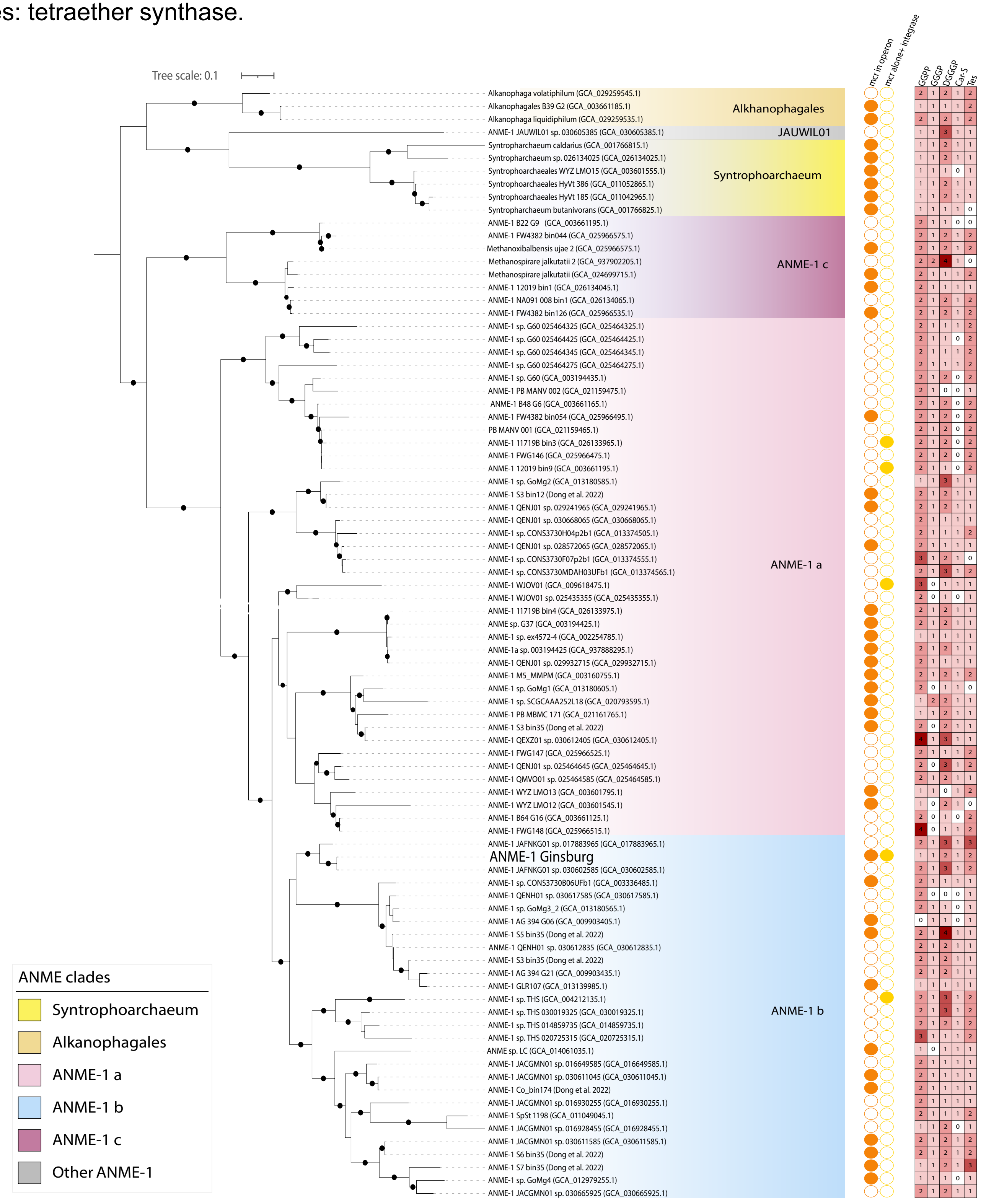

Supplement: Figure_S1_ycag072 [file figure_s1_ycag072.pdf]
